# Berbamine hydrochloride potently inhibits SARS-CoV-2 infection by blocking S protein-mediated membrane fusion

**DOI:** 10.1371/journal.pntd.0010363

**Published:** 2022-04-25

**Authors:** Zhe-Rui Zhang, Ya-Nan Zhang, Hong-Qing Zhang, Qiu-Yan Zhang, Na Li, Qi Li, Cheng-Lin Deng, Bo Zhang, Xiao-Dan Li, Han-Qing Ye

**Affiliations:** 1 Key Laboratory of Special Pathogens and Biosafety, Wuhan Institute of Virology, Center for Biosafety Mega-Science, Chinese Academy of Sciences, Wuhan, China; 2 University of Chinese Academy of Sciences, Beijing, China; 3 Drug Discovery Center for Infectious Disease, Nankai University, Tianjin, People’s Republic of China; 4 Hunan Normal University, School of Medicine, Changsha, China; Minia University, EGYPT

## Abstract

COVID-19 caused by SARS-CoV-2 has posed a significant threat to global public health since its outbreak in late 2019. Although there are a few drugs approved for clinical treatment to combat SARS-CoV-2 infection currently, the severity of the ongoing global pandemic still urges the efforts to discover new antiviral compounds. As the viral spike (S) protein plays a key role in mediating virus entry, it becomes a potential target for the design of antiviral drugs against COVID-19. Here, we tested the antiviral activity of berbamine hydrochloride, a bis-benzylisoquinoline alkaloid, against SARS-CoV-2 infection. We found that berbamine hydrochloride could efficiently inhibit SARS-CoV-2 infection in different cell lines. Further experiments showed berbamine hydrochloride inhibits SARS-CoV-2 infection by targeting the viral entry into host cells. Moreover, berbamine hydrochloride and other bis-benzylisoquinoline alkaloids could potently inhibit S-mediated cell-cell fusion. Furthermore, molecular docking results implied that the berbamine hydrochloride could bind to the post fusion core of SARS-CoV-2 S2 subunit. Therefore, berbamine hydrochloride may represent a potential efficient antiviral agent against SARS-CoV-2 infection.

## Author summary

The recent COVID-19 pandemic has caused a global health threat. Development of antiviral agents are urgently required to control the pandemic. In this study, we evaluated the antiviral activity of berbamine hydrochloride, a bis-benzylisoquinoline alkaloid that is isolated from traditional Chinese medicinal Berberis amurensis. We demonstrated that berbamine hydrochloride potently inhibits SARS-CoV-2 infection by blocking the entry of the virus into the host cell. Moreover, berbamine hydrochloride and other bis-benzylisoquinoline alkaloids also block SARS-CoV-2 S-mediated membrane fusion. Furthermore, we analyzed the interaction of berbamine hydrochloride and viral proteins by molecular docking analysis. We found that berbamine hydrochloride could directly bind to the post fusion core of SARS-CoV-2 S2 subunit, which mediates the fusion with the host membranes. Our results provide a new lead compound for antiviral agents against SARS-CoV-2 infection.

## Introduction

Coronavirus disease 2019 (COVID-19) is a respiratory illness caused by an emerging novel coronavirus SARS-CoV-2. Since the outbreak at the end of 2019, the COVID-19 pneumonia pandemic spreads rapidly around the world and causes a serious global social crisis [1]. Most recently, there are a few drugs approved for clinical treatment to combat SARS-CoV-2 infection. Molnupiravir, a potent RNA-dependent RNA polymerase inhibitor, is the first approved oral antiviral agent for treating COVID-19. Ritonavir in combination with PF-07321332 was approved for emergency use by the US Food and Drug Administration on 22 December 2021 for the treatment of mild-to-moderate COVID-19 disease [2]. However, the severity of the ongoing global pandemic still urges the efforts to discover new antiviral compounds.

SARS-CoV-2 belongs to the genus *Betacoronavirus* and is an enveloped, single-stranded positive-sense RNA virus with a genome size of ∼30 kb [3]. The SARS-CoV-2 genome contains multiple functional open reading frames (ORFs) that code replicase (ORF1a/ORF1b), spike (S), envelope (E), membrane (M) and nucleocapsid (N), respectively [4]. The S protein, residing on the virion surface as trimers, plays a key role in virus entry [5]. It consists of two subunits: S1 and S2. The S1 subunit contains the receptor-binding domain (RBD), which is involved in host cell receptor binding, and the S2 subunit is responsible for the fusion of viral and cellular membranes [5–9]. In infected cells, the encoded S protein is trafficked to the cell surface and induces cell-cell membrane fusion and the formation of multinucleated cells (syncytia), which is believed to be important for both pathogenicity and transmission of the virus [10]. The presence of infected multinucleated syncytial pneumocytes has been demonstrated to be associated with severe cases of COVID-19 [10]. Therefore, to explore membrane fusion inhibitors is very useful to block virus pathogenicity and transmission.

Natural compounds, especially the ones derived from plants, has played a crucial role in drug discovery due to their potential multiple targets, bioactivities and limited toxicity [11]. Over the past three decades, more than 50% of approved drugs and drug candidates are natural compounds or derivatives thereof [12,13]. As such, the natural compounds provide a rich basis for the discovery and development of anti-SARS-CoV-2 drugs.

Berbamine hydrochloride is a bis-benzylisoquinoline alkaloid that is isolated from traditional Chinese herbal medicine Berberis amurensis. At present, some studies have reported the inhibitory effects of berbamine hydrochloride on SARS-CoV-2 *in vitro*. By in silico analysis, berbamine hydrochloride was demonstrated to be able to bind to ACE2, GRP78 and TMPRSS2 of the host cells [14] and have high affinity with the main protease (M^pro^) of SARS-CoV-2 [15], respectively. However, these findings have not been further verified in experiments. Sangeun Jeon *et al* carried out a high content chemical screening using an FDA-approved drug library and discovered that berbamine hydrochloride showed potential antiviral activities against SARS-CoV-2 [16]. Lihong Huang *et al* showed that berbamine inhibits SARS-CoV-2 infection by compromising TRPMLs-mediated endolysosomal trafficking of ACE2 [17].

Here, we found a new antiviral mechanism for berbamine hydrochloride against SARS-CoV-2 by blocking virus entry through inhibiting the spike protein (S) -mediated cell-cell fusion. Moreover, other bis-benzylisoquinoline alkaloids (including tetrandrine, liensinine, isoliensinine, fangchinoline, and cepharanthine) also block S-mediated membrane fusion. Molecular docking analysis implied that the berbamine hydrochloride could bind to the post fusion core of SARS-CoV-2 S2 subunit. Consequently, we demonstrated that berbamine hydrochloride may be useful as lead compound for antiviral agents against SARS-CoV-2 infection.

## Materials and methods

### Cells, viruses, antibodies and reagents

Vero-E6 cells (ATCC CRL-1686) and Caco2 cells (ATCC HTB-37) were kindly provided by Prof. Zheng-Li, Shi (Wuhan institute of Virology, CAS). BHK-21 cells *were* kindly provided by Prof. Han-Zhong, Wang (Wuhan institute of Virology, CAS). For all cell culture procedures, the cells were cultivated in Dulbecco’s modified Eagle Medium (DMEM; Invitrogen, Darmstadt, Germany) supplemented with 10% fetal bovine serum (FBS; Gibco), and penicillin (100 U/ml)-streptomycin (100 μg/ml) at 37°C with 5% CO_2_. SARS-CoV-2 (WIV-04) (3) was propagated in Vero E6 cells and stored in aliquots at −80°C for experiments. All the experiments using the live SARS-CoV-2 virus were performed in a BSL-3 laboratory at Wuhan Institute of Virology according to standard BSL-3 guidelines. The antibody against the NP protein of RP3-CoVwas kindly provided by Prof. Zheng-Li, Shi (Wuhan institute of Virology, CAS), which is cross-reactive with the NP protein of SARS-CoV-2. FITC-conjugated goat anti-rabbit IgG was purchased from Protein Tech Group. Berbamine hydrochloride, liensinine, isoliensinine, tetrandrine, fangchinoline and cepharanthine were purchased from Weikeqi Biotech (Sichuan, China).

### Plaque assay

The titer of SARS-CoV-2 virus stock was determined by plaque assay. Briefly, virus supernatants were 10-fold serially diluted, and were added into confluent Vero E6 cells seeded in 24-well plate (plated 1 day in advance). Infected cells were incubated at 37°C for 1 h before medium containing 1% methylcellulose was overlaid. After incubation for 4 days at 37°C with 5% CO_2_, the cells were fixed with 3.7% formaldehyde and stained with 1% crystal violet. The viral titer was calculated as plaque forming units (PFU)/mL.

### Real-time RT-PCR

Viral RNAs were extracted from the supernatant of the infected cells using QIAamp viral RNA mini kit (52906, Qiagen) following the manufacturer’s protocol. Real-time RT-PCR was performed using Universal Probe One-Step RT-PCR Kit (catalog no. E3006; NEB). For genome RNA quantification, the primer pair (RBD-qF1: 5’- CAATGGTTTAACAGGCACAGG-3’, RBD-qR1: 5’- CTCAAGTGTCTGTGGATCACG-3’ and Probe: ACAGCATCAGTAGTGTCAGCAATGTCTC) were used. The amplification condition for real-time RT-PCR assay consists of RT step at 42°C for 15min, RT inactivation at 95°C for 60s, followed by 40 cycles of PCR at 95°C for 10s (denaturation) and 60°C for 45s (annealing and extension).

### Cytotoxicity assays

The cytotoxicity of the berbamine hydrochloride on Vero E6 and Caco2 cells were determined by CCK8 assays according to the manufacturer’s protocols, which performed in BSL-2 laboratory. Briefly, Vero-E6 cells and Caco2 cells were seeded in 96-well plates (1×10^4^ cells per well). After one day cultivation, cells were treated with concentration gradient of berbamine hydrochloride for 36 h, and for each drug concentration, two wells were performed in parallel. Subsequently, the cells were incubated with 10 μL CCK8 reagent (1:100; cell counting kit-8, Bimake) for 1 h at 37°C. The absorbance at 450 nm was measured by a multimode Microplate Reader (Varioskan Flash, Thermo Fisher). Cell activity was expressed as the percentage of the absorption value of the treated cells to the untreated cells. For each concentration, mean values of the cell viability were calculated. The half-maximal cytotoxicity concentration (CC_50_) was calculated by nonlinear regression using GraphPad Prism 8.0.

### *In vitro* antiviral assays

To evaluate the antiviral efficacy of the drug, we performed antiviral assay. Briefly, Vero E6 and Caco2 cells were infected with the SARS-CoV-2 at an MOI of 0.01. The infected cells were treated with different concentrations of berbamine hydrochloride, tetrandrine, fangchinoline and cepharanthine, respectively. At 36 h p.i., the amounts of infectious viruses in culture fluids were quantified by real time RT-PCR assay. The antiviral activity of the compounds was expressed as 50% effective concentration (EC_50_) and was calculated with the GraphPad Prism software 8.0 using the nonlinear regression analysis.

### Time of addition assay

The time of addition experiment was used to test the drug inhibition stage of the SARS-CoV-2 life cycle. Vero E6 cells were treated with berbamine hydrochloride or DMSO at the following time points: full-time-infection (−1 to 12 h), during-time-infection (0–2 h), and post-time-infection (2–12 h). For “Full-time-infection” treatment, Vero E6 cells were pre-treated with the drugs for 1 h and the virus was applied for 2 h to allow infection. Then, the virus-drug mixture was removed. Cells were washed with PBS, and further cultured with drug-containing medium until the end of the experiment. For “Entry” treatment, the drugs were added to the cells for 1 h before virus infection, and maintained during the 2 h viral attachment process. Then, the virus-drug mixture was replaced with fresh culture medium without drugs till the end of the experiment. For “Post-entry” experiment, virus was added to the cells to allow infection for 2 h, and then virus-containing supernatant was replaced with drug-containing medium until the end of the experiment. The DMSO-treatment group of the final selected studies was consistent with that of the “Full-time” group. For all the experimental groups, cells were infected with virus at an MOI of 0.05, and at 12 h p.i., the amounts of infectious viruses in culture fluids were quantified by real time RT-PCR assay. Inhibition rate of berbamine hydrochloride was calculated as the percentage of viral RNA copies relative to DMSO control.

### Transient replicon assay

A *Renilla* luciferase (Rluc) replicon of SARS-CoV-2, which was constructed in our laboratory and could be operated in BSL-2 lab to represent virus transcription and replication [18], was used to determine the inhibitory effect of berbamine hydrochloride on SARS-CoV-2 RNA replication. BHK-21 cells were electroporated with 10 μg of replicon RNA using a GenePulser Xcell system (Bio-Rad, Hercules, CA) according to an established protocol. The transfected cells were seeded in 12-well plate, simultaneously treated with 10 μM berbamine hydrochloride or DMSO. At different time points post transfection (p.t.), the cells were washed once with PBS and lysed with 200 μL lysis buffer (Promega). 20 μL of cell lysates were used to determine for luciferase signals after adding 50 μL substrate. The antiviral efficacy of berbamine hydrochloride against SARS-CoV-2 RNA replication was evaluated by the reduction of luciferase signals. DMSO is the solvent for the compounds and is used as a control.

### SARS-CoV-2 Spike-mediated pseudovirus entry assay

Vero-E6 cells were seeded in a 12- well plate for 24 h, and then were treated with 10 μM berbamine hydrochloride. After incubation for 2 h, the cells were infected with VSV-delG-Rluc-SARS-CoV-2-S pseudovirus. At 24 h post-infection, cells were lysed and the intracellular luciferase activities were measured according to the manufacturer’s protocols. Experiments were performed at least twice in BSL-2 laboratory.

### Virus attachment assay

Vero E6 cells was infected with SARS-CoV-2 (MOI = 0.05) and incubated at 4°C for 1 h to allow virus attachment in the presence of berbamine hydrochloride (10 μM) or DMSO control. After synchronized adsorption for 1 h, the unbound SARS-CoV-2 virions were removed by PBS washing, and the relative levels of bound SARS-CoV-2 virions were measured by qRT-PCR assay.

### Syncytium formation inhibition assay

BHK-21 cells were transfected with 2 μg plasmid pCAGGS-SARS-CoV-2-S encoding the SARS-CoV-2 S protein and cultured in DMEM containing 10% FBS at 37°C for 24 h. Vero E6 cells (5×10^4^ cells per well) were incubated in 12-well plates at 37°C for 24 h, followed by the addition of 5×10^4^ BHK-21/SARS-CoV-2/S cells, in the absence or presence of 10 μM berbamine hydrochloride, liensinine, isoliensinine, tetrandrine, fangchinoline and cepharanthine. After co-culture at 37°C for 12 h, the fused or unfused Vero E6 cells were counted under an inverted fluorescence microscope.

### Immunofluorescence analysis

Vero E6 cells were fixed with cold 5% acetone in methanol for 10 min at room temperature. Cells were then incubated with primary antibodies (rabbit anti-SARS-CoV-2 NP protein antibody) overnight at 4°C. After washing three times with PBS, the cells were incubated with FITC-conjugated goat anti-rabbit polyclonal secondary antibody for 40 min at room temperature. After washing three times with PBS, the cells were incubated with 4, 6-diamidino-2-phenylindole (DAPI) for nuclear counterstaining for 5 min at room temperature. Images were captured under a fluorescent microscope (Nikon Eclipse TE2000).

### Molecular docking

In order to further verify the potential drug targets, ligand based molecular docking between berbamine hydrochloride and the post fusion core of SARS-CoV-2 S2 subunit were performed using AutoDock Vina. The 3D structure of berbamine hydrochloride was obtained from ZINC (ID: ZINC38139356). The 3D structure of the post fusion core of SARS-CoV-2 S2 subunit was obtained from PDB (ID: 6LXT) databases. The resulting docking poses and affinity scores (in kcal/mol) were given by AutoDock Vina. The figure was drawn using PyMOL Molecular Graphics System.

### Statistical analyses

Statistical analyses were performed using GraphPad Prism 8 software. An unpaired two-tailed student’s t-test was used to analyze the statistical significance of two groups.

## Results

### Berbamine hydrochloride inhibits SARS-CoV-2 infection in different cell lines

**Fig 1:**
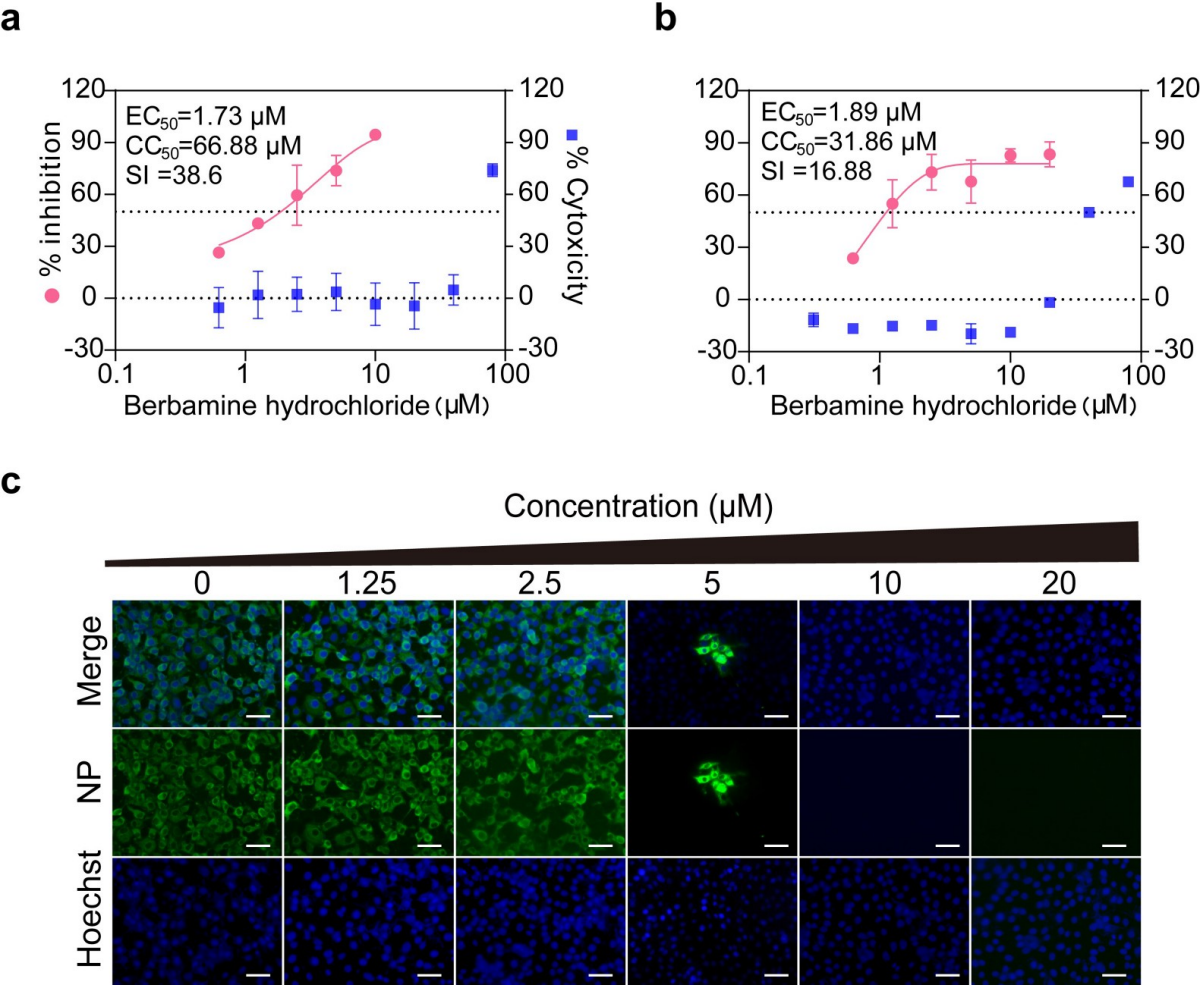
Antiviral activity of Berbamine hydrochloride against SARS-CoV-2 *in vitro*. Vero-E6 (a) or Caco2 (b) cells were infected with SARS-CoV-2. After incubation with berbamine hydrochloride at 37°C for 36 h, cell culture fluids were harvested for qRT-PCR assay. Cytotoxicity was examined by CCK-8 assay. The CC_50_, IC_50_, and SI values for each inhibitor are shown above the figures. Error bars represents standard deviation. (c) Immunofluorescence assay of SARS-CoV-2-infected Vero E6 cells that received berbamine hydrochloride treatment. Scale bar, 100 μm. Data was expressed as mean ± standard deviation and shown are representative results of two independent experiments with two technical replicas per experiment.

In the present study, we first evaluated the antiviral activity of berbamine hydrochloride against SARS-CoV-2 infection using different cell lines. As demonstrated in Fig 1A and 1B, berbamine hydrochloride effectively inhibited SARS-CoV-2 infection in both Vero E6 and Caco2 cells in a dose-dependent manner with EC_50_ values of 1.732 and 1.887 μM, respectively. In contrast, the CC_50_ values measured by CCK8 assay in Vero E6 and Caco2 cells were 66.88 and 31.86 μM, and the selective index (SI) were 38.6 and 16.88, respectively. Meanwhile, we also detected the expression levels of viral N protein in SARS-CoV-2-infected Vero E6 cells by immunofluorescence assay (IFA) upon treatment with berbamine hydrochloride (Fig 1C). Consistently, a dose-dependent reduction in the percentage of IFA-positive cells was observed. These results indicated that berbamine hydrochloride effectively inhibited SARS-CoV-2 infection in different cell lines.

### Berbamine hydrochloride inhibits SARS-CoV-2 infection by targeting the stage of viral entry

**Fig 2:**
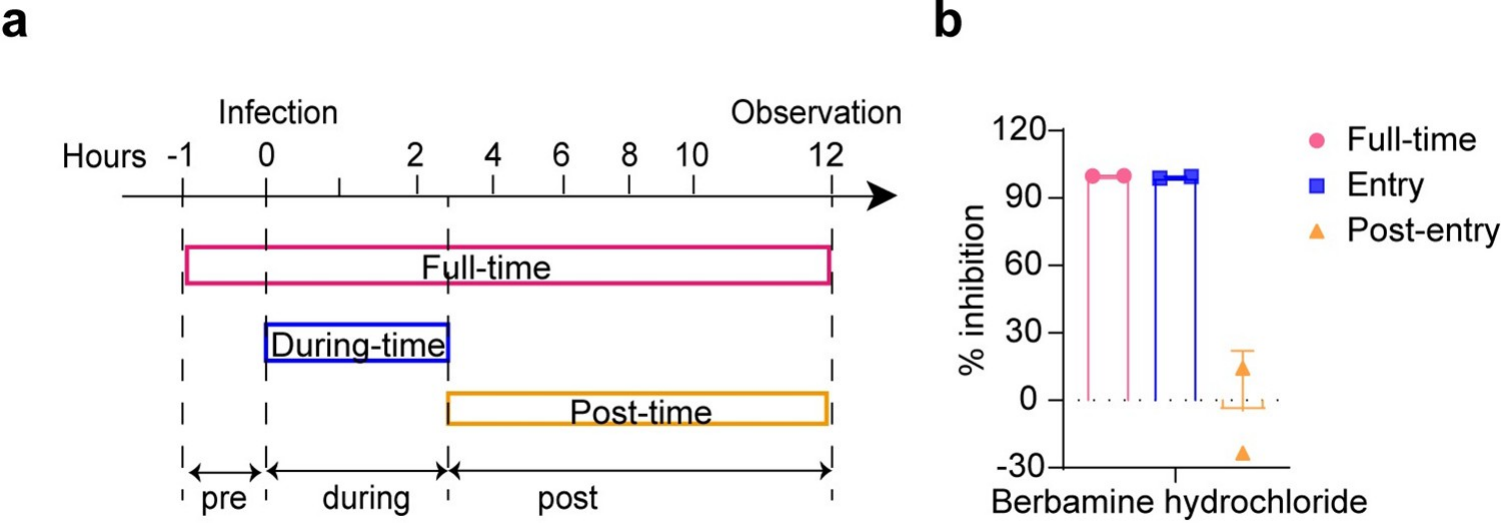
Time-of-addition analysis. (a) The schematic diagram of time-of addition. Approximately 8×10^4^ Vero-E6 cells were seeded per well of a 24-well plate. After incubation for 24 h, the cells were infected with the SARS-CoV-2 virus at an MOI of 0.05. Berbamine hydrochloride was added to the infected cells at a final concentration of 10 μM at indicated time points. At 12 h p.i., the cell culture fluids were harvested for qRT-PCR assay (b). Data was expressed as mean ± standard deviation and shown are representative results of two independent experiments with two technical replicas per experiment. Error bars represents standard deviation.

To investigate the inhibitory stage of berbamine hydrochloride in SARS-CoV-2 life cycle, a time-of-addition assay was performed. Based on the single-cycle replication of the virus in Vero-E6 cells, a schematic diagram of the experimental design is shown in Fig 2A. Vero E6 cells were treated with berbamine hydrochloride or DMSO at the following time points: full-time-infection, the drug was present throughout the entire time of viral infection (−1 to 12 h); during-time-infection, the drug was present at the early stages of SARS-CoV-2 infection (0–2 h); and post-time-infection, the drug was present at the post stages of SARS-CoV-2 infection (2–12 h). As indicated in Fig 2B, berbamine hydrochloride significantly reduced the copy numbers of viral RNA in culture fluids with full-time-infection and during-time-infection treatment compared to the DMSO control. However, there was no significant differences between the post-time-infection and DMSO treatment. The above results imply that berbamine hydrochloride acts at the early stages of SARS-CoV-2 infection.

**Fig 3:**
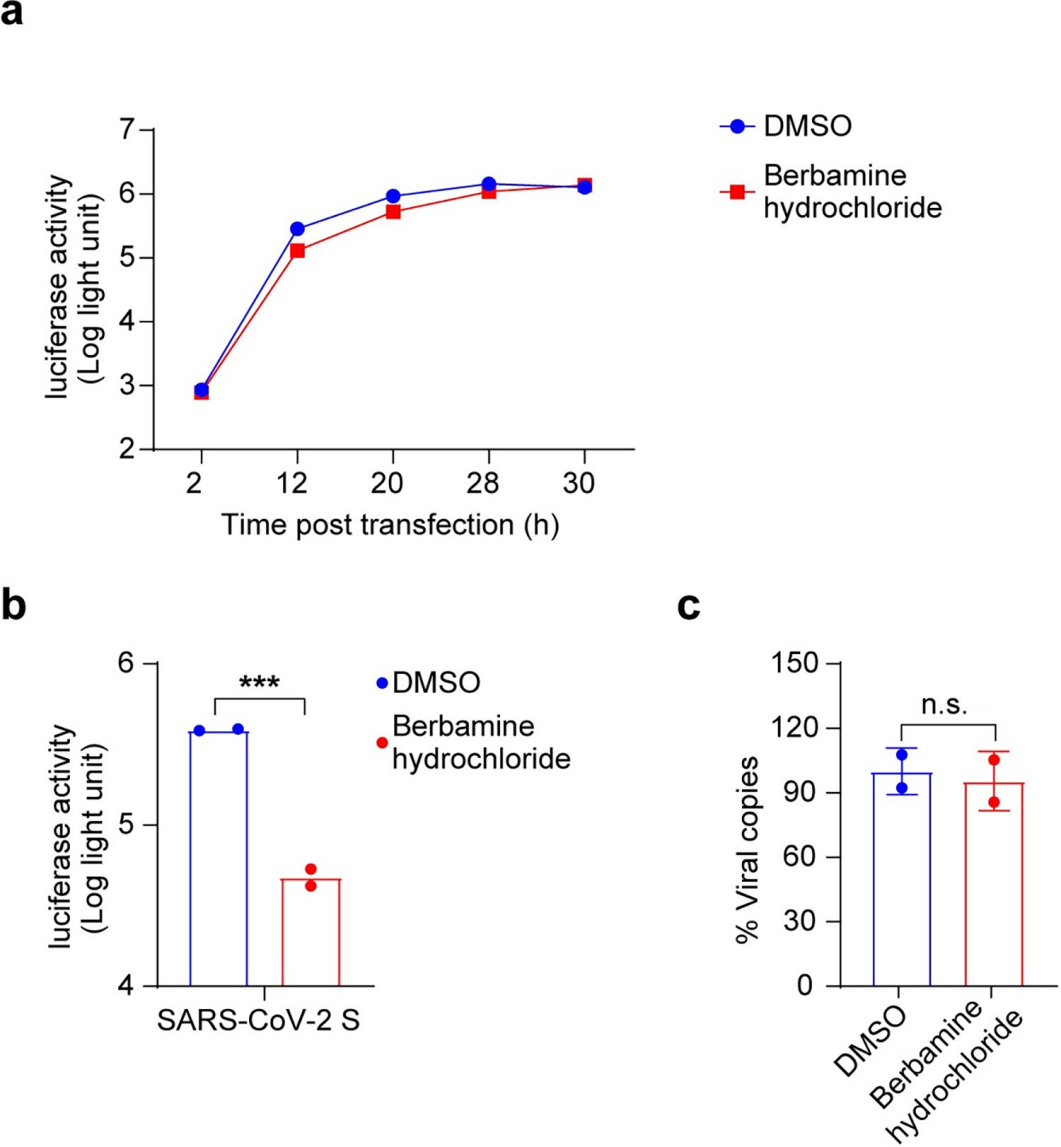
Mode-of-action analysis. (a) Transient replicon assay. (b) SARS-CoV-2 pseudovirus assay. Vero E6 cells were treated with the berbamine hydrochloride (10 μM) or DMSO, and then infected with SARS-CoV-2 pseudovirus for 24 h. Luciferase activity in cell lysates was determined and compared with the DMSO control. (c) Impact of berbamine hydrochloride on SARS-CoV-2 attachment. Vero E6 cells were treated with berbamine hydrochloride (10 μM) or DMSO for 1 h prior to SARS-CoV-2 infection at 4°C for 1 h. The cells containing binding virions were collected for quantification of viral RNA copies by real-time RT-PCR assay. Data was expressed as mean ± standard deviation and shown are representative results of two independent experiments with two technical replicas per experiment. An unpaired two-tailed student’s t-test was performed for statistical analysis. n.s. not significant, *p<0.05, **p<0.01 and ***p<0.001. Error bars represents standard deviation.

Then, the effects of berbamine hydrochloride on the early events of virus infection, including viral entry, genome transcription and replication, were evaluated. The results of the transient replicon assay showed that there was no significant difference between DMSO and drug treatment, excluding the function of berbamine hydrochloride in the stages of viral genome transcription and replication (Fig 3A). The inhibitory effects of berbamine hydrochloride on virus entry stage was evaluated using a vesicular stomatitis virus (VSV)-based reporter pseudovirus that expresses the spike protein of SARS-CoV-2. At 24 h post pseudovirus infection, the cells were lysed and the activity against SARS-CoV-2 entry was tested via quantification of luciferase activities in cell lysates. As shown in Fig 3B, berbamine hydrochloride exhibited direct inhibitory effect on pseudovirus entry and the intracellular luciferase activity was reduced by about 10-fold compared to DMSO treatment. These results indicate that berbamine hydrochloride mainly inhibits viral entry.

It has been reported that the entry of SARS-CoV-2 into host cells is mediated by the binding of viral S protein to the host ACE2 receptor. Hence, we examined whether berbamine hydrochloride affects the attachment of SARS-CoV-2 to cells. As shown in Fig 3C, in the viral attachment assay, there was no significant difference of cell-attached viral RNA copies between the berbamine hydrochloride treatment group and the DMSO control group. Therefore, the blockade of virus entry by berbamine hydrochloride is not related to the attachment of SARS-CoV-2 to cells.

### Berbamine hydrochloride potently inhibit S-mediated cell-cell fusion

**Fig 4:**
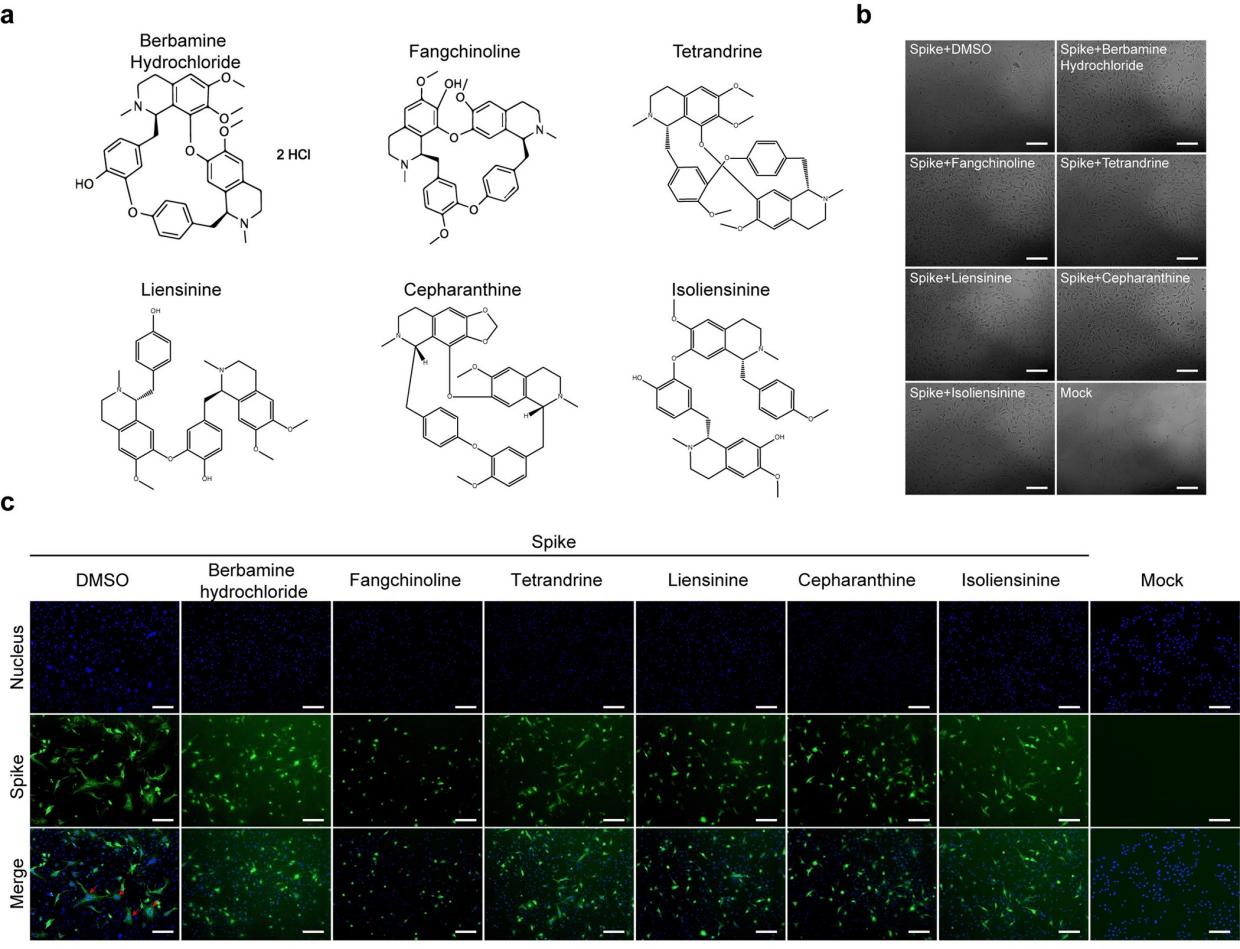
Microscopic observation and immunofluorescence analysis the inhibitory activity of bis-benzylisoquinoline alkaloids against SARS-CoV-2 S-mediated cell–cell fusion. (a) Structure of bis-benzylisoquinoline alkaloids. Vero-E6 cells co-culture with BHK that transfected with the SARS-CoV-2 S expression construct and treated with 10 μM berbamine hydrochloride, liensinine, isoliensinine, tetrandrine, fangchinoline, cepharanthine or DMSO. Cells were harvested at 12 h after treatment. The syncytial phenotype is indicated by the multinucleated giant cells as indicated by the red arrows. (b) Microscopic observation and (c) immunofluorescence images of cells staining with DAPI (blue) or anti-S Ab (green). Scale bar, 100 μm. Experiments were performed at least twice with two technical replicas per experiment.

After virus attachment to host receptor, the next SARS-CoV-2 S protein-mediated membrane fusion process plays an important role in viral entry. Hence, we examined whether berbamine hydrochloride could block S protein-mediated cell fusion. BHK cells overexpressing SARS-CoV-2 S protein were used as the effector cells and Vero E6 cells were used as the target cells. As showed in Fig 4B and 4C, when the effector and target cells were cocultured, the DMSO group induced an extensive syncytial phenotype with fused cells containing multiple nuclei, whereas little or no such syncytial phenotype was observed in cells treated with berbamine hydrochloride. Taken together, berbamine hydrochloride potently inhibits the S-mediated cell-cell fusion.

In our previous study, the bis-benzylisoquinoline alkaloids including liensinine, isoliensinine, tetrandrine, fangchinoline and cepharanthine were found to efficiently inhibit the SARS-CoV-2 induced cytopathic effect [19]. However, the specific inhibitory effect on SARS-CoV-2 infection of tetrandrine, fangchinoline and cepharanthine were not identified. Therefore, in this study, to confirm their antiviral activity against SARS-CoV-2, we further tested the EC_50_ and CC_50_ of tetrandrine, fangchinoline and cepharanthine, respectively. As shown in S1 Fig, tetrandrine, fangchinoline and cepharanthine exhibited a dose-dependent inhibition against SARS-CoV-2 infection. Compared to that of berbamine hydrochloride, those bis-benzylisoquinoline alkaloids showed slightly weaker antiviral activity (S1 Table). The underlying antiviral mechanisms of these bis-benzylisoquinoline alkaloids remain unknown. The chemical structures of these drugs are displayed in Fig 4A. To probe the structure-activity relationship of such bis-benzylisoquinoline alkaloids as antiviral agents for SARS-CoV-2, these bis-benzylisoquinoline alkaloids were tested for their capability to inhibit S-mediated cell-cell fusion as berbamine hydrochloride did. As shown in Fig 4B and 4C, apparent inhibitory effects on S-mediated cell-cell fusion were also observed for these compounds, implying that those bis-benzylisoquinoline alkaloids have a common antiviral effect by interfering the S-mediated cell-cell fusion.

### Berbamine hydrochloride could bind to the post fusion core of SARS-CoV-2 S2 subunit

**Fig 5:**
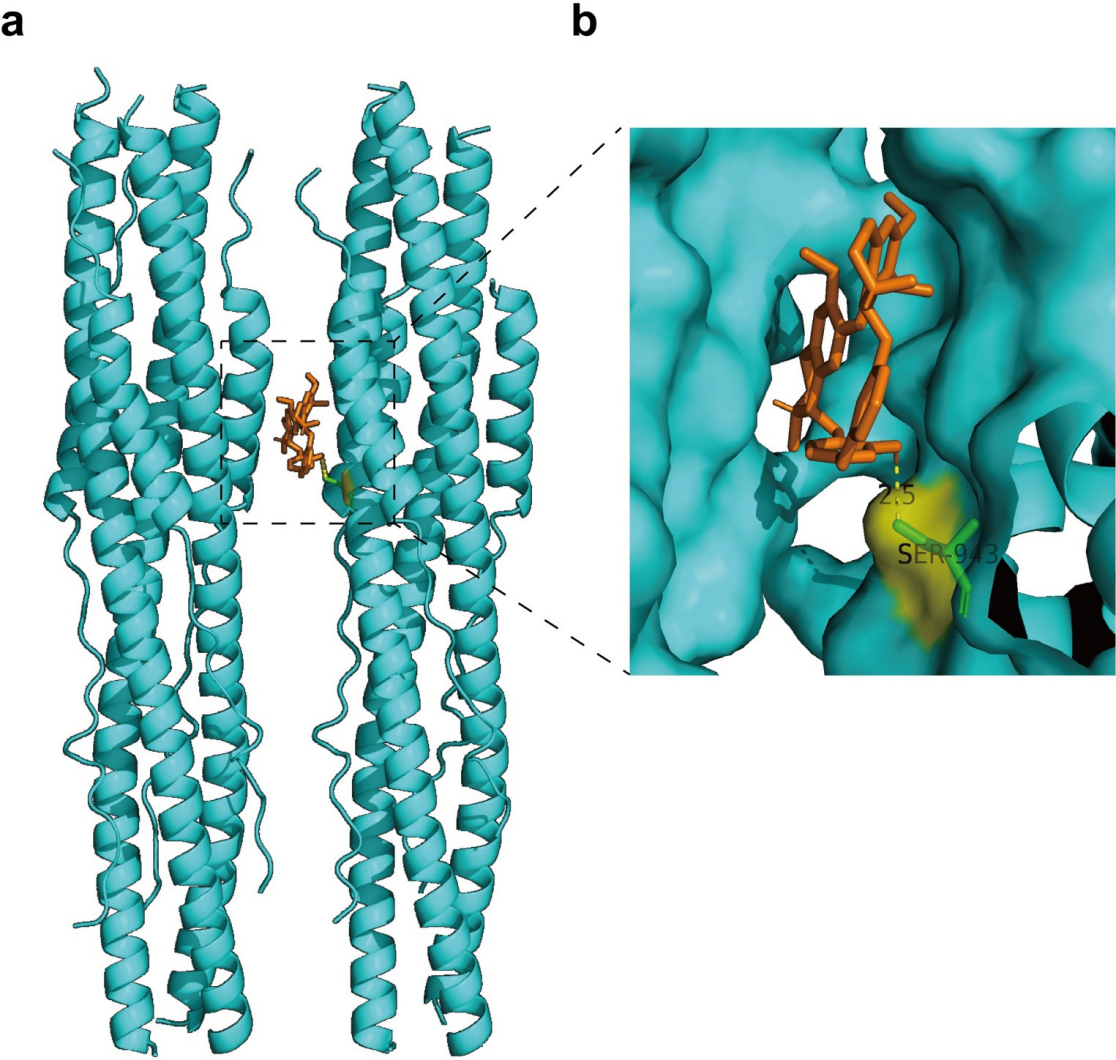
Generated the binding mode of berbamine hydrochloride in the post fusion core of SARS-CoV-2 S2 subunit. The 3D structure of berbamine hydrochloride and proteins were obtained from ZINC (ID: ZINC38139356) and PDB (ID: 6LXT) databases, respectively. The interaction sites between berbamine hydrochloride and S2 were predicted by Autodock vina algorithm. The SARS-CoV-2 S2 residues involved in berbamine hydrochloride binding are shown as green sticks.

SARS-CoV-2 S2 subunit play a key role in the fusion of viral and cellular membranes [20]. In order to analyze other potential mechanisms of berbamine hydrochloride against SARS-CoV-2 infection, we performed molecular docking using the reported structures of post fusion core of SARS-CoV-2 S2 subunit with Autodock vina. The 3D structure of berbamine hydrochloride and proteins were obtained from ZINC and PDB databases, respectively. The interaction sites between berbamine hydrochloride and S2 were predicted by Autodock vina algorithm. As indicated in Fig 5A, berbamine hydrochloride could directly bind to the post fusion core of SARS-CoV-2 S2 subunit and the docking scores were -9.6 kcl/mol. Further analysis revealed that berbamine hydrochloride could directly bind to S2 subunit with the residue S943 (Fig 5B). Therefore, we speculated that berbamine hydrochloride potentially inhibits SARS-CoV-2 S protein-mediated membrane fusion by blocking the fusion activity of S2 subunit.

## Discussion

Since its outbreak at the end of 2019, the COVID-19 has become the most severe threat to global health. Although some antiviral agents approved for clinical treatment to combat SARS-CoV-2 infection currently, it is still urgent to identify efficient antiviral inhibitors to control the current SARS-CoV-2 pandemic.

Natural product-based medicines are a class of low toxicity and highly reactive compounds [21,22]. The World Health Organization encourages developing natural product-based medicines against human diseases [23]. Thus, it is worth searching for natural product-based medicines for the treatment of COVID-19. Berbamine hydrochloride, which is a natural bis-benzylisoquinoline alkaloid found in a variety of herbs, has been widely used in clinical treatment of leukopenia in China for many years and the usual dose in adults is 112 mg administered orally three times per day [24]. Besides, clinical studies in humans have substantiated that berbamine hydrochloride has multiple benefits to health including potential diabetes treatment, antihypertensive, protection against cognitive decline, anti-inflammatory and anti-cancer effects, with outstanding safety profile, low toxicity, and fewer minor side effects after oral administration [25]. Therefore, repurposing of berbamine hydrochloride to inhibit SARS-CoV-2 represents a great opportunity to be quickly applied to clinical antiviral therapy against COVID-19.

In this study, we found that berbamine hydrochloride efficiently inhibited SARS-CoV-2 infection by interfering with the entry stage but not the genome replication of the viral life cycle. Moreover, syncytium formation inhibition assay suggested that the berbamine hydrochloride specifically inhibits syncytium formation by blocking S-mediated membrane fusion. In addition, some other bis-benzylisoquinoline alkaloids which showed antiviral activity against SARS-CoV-2 also efficiently inhibited S-mediated syncytium formation. These results demonstrate that the S mediated membrane fusion is an important target for the antiviral activities of these bis-benzylisoquinoline alkaloids.

Furthermore, molecular docking analysis implied that the berbamine hydrochloride could bind to the post fusion core of SARS-CoV-2 S2 subunit with the residue S943. A recent study has shown that the spike-S943 could enhance the interaction between HR1 and HR2 to stabilize the structure of S2 subunit and promote membrane fusion [20]. Therefore, there is a possibility that, by binding with the significant S943 site within S2 subunit, berbamine hydrochloride blocks the interaction between the HR1 and HR2 domains, leading to the inhibitory effect on membrane fusion.

In addition, berbamine hydrochloride has been reported to have various regulatory functions on cells. Berbamine hydrochloride is a trypsin-like serine protease inhibitor [26]. Previous studies have shown that the cleavage of S protein by host proteases is a prerequisite for virus-cell membrane fusion [27]. The transmembrane protease serine protease-2, which plays important role in S protein cleavage and virus entry, belongs to trypsin-like protease. Therefore, there is a possibility that berbamine hydrochloride may have inhibitory effect on the cleavage of S proteins, leading to the entry defect of the virus. In addition, berbamine hydrochloride had been reported as calcium channel blocker [28]. There are several evidences in the literature showing that SARS-CoV-2 infection induces calcium release of the infected cells [29], and the compound nicosamide, which inhibits the calcium-activated ion channel TMEM16F, could efficiently block SARS-CoV-2 S-induced syncytia and virus infection [30]. Thus, as a calcium channel inhibitor, berbamine hydrochloride may have a similar antiviral mechanism against SARS-CoV-2 by blunting the calcium oscillations caused by virus infection. We will verify these speculations in future work to further explain the antiviral mechanism of berbamine hydrochloride against SARS-CoV-2.

Berbamine hydrochloride currently is being used clinically as anti-inflammatory agent. Therefore, berbamine hydrochloride might have a potential effect on the inhibition of cytokine storm in the lungs of patients induced by SARS-CoV-2. However, due to resource limitations, we were unable to test the antiviral activity of berbamine hydrochloride against SARS-CoV-2 infection *in vivo* in this study. The protection effects of berbamine hydrochloride on SARS-CoV-2 would be further detected in different animal models in our future work.

In summary, we explored the antiviral activity of berbamine hydrochloride against SARS-CoV-2 infection *in vitro* and demonstrated that the spike-mediated membrane fusion is one of the targets for its antiviral activity. Taken together, our study suggests that berbamine hydrochloride may be a potential antiviral agent for the treatment of COVID-19.

## Supporting information

S1 Fig Antiviral activity of tetrandrine, fangchinoline and cepharanthine against SARS-CoV-2 *in vitro*. Vero E6 cells were infected with SARS-CoV-2 (MOI = 0.01). After incubation with different concentrations of tetrandrine (a), fangchinoline (b) and cepharanthine (c) at 37°C for 36 h, cell culture fluids were harvested for real-time RT-PCR assay. Cytotoxicity was examined by CCK-8 assay. The CC_50_, IC_50_, and SI values for each inhibitor are shown. Experiments were performed at least twice with two technical replicas per experiment and the data are presented as the mean ± standard deviation. Error bars represents standard deviation.
(TIF)

S1 Table The CC_50_, IC_50_, and SI values for Berbamine hydrochloride, tetrandrine, fangchinoline and cepharanthine against SARS-CoV-2 *in vitro*. (DOCX)

S1 Data The numerical data used in all figures. Excel spreadsheet containing, in separate sheets, the underlying numerical data and statistical analysis for Figs 1A, 1B, 2B, 3A, 3B, 3C, S1A, S1B and S1C.
(XLSX)
