# Supplementary figures and images for: Berbamine hydrochloride potently inhibits SARS-CoV-2 infection by blocking S protein-mediated membrane fusion
Source: PLoS Negl Trop Dis. 2022 Apr 25;16(4):e0010363. doi: 10.1371/journal.pntd.0010363 (PMC9071123; doi:10.1371/journal.pntd.0010363)

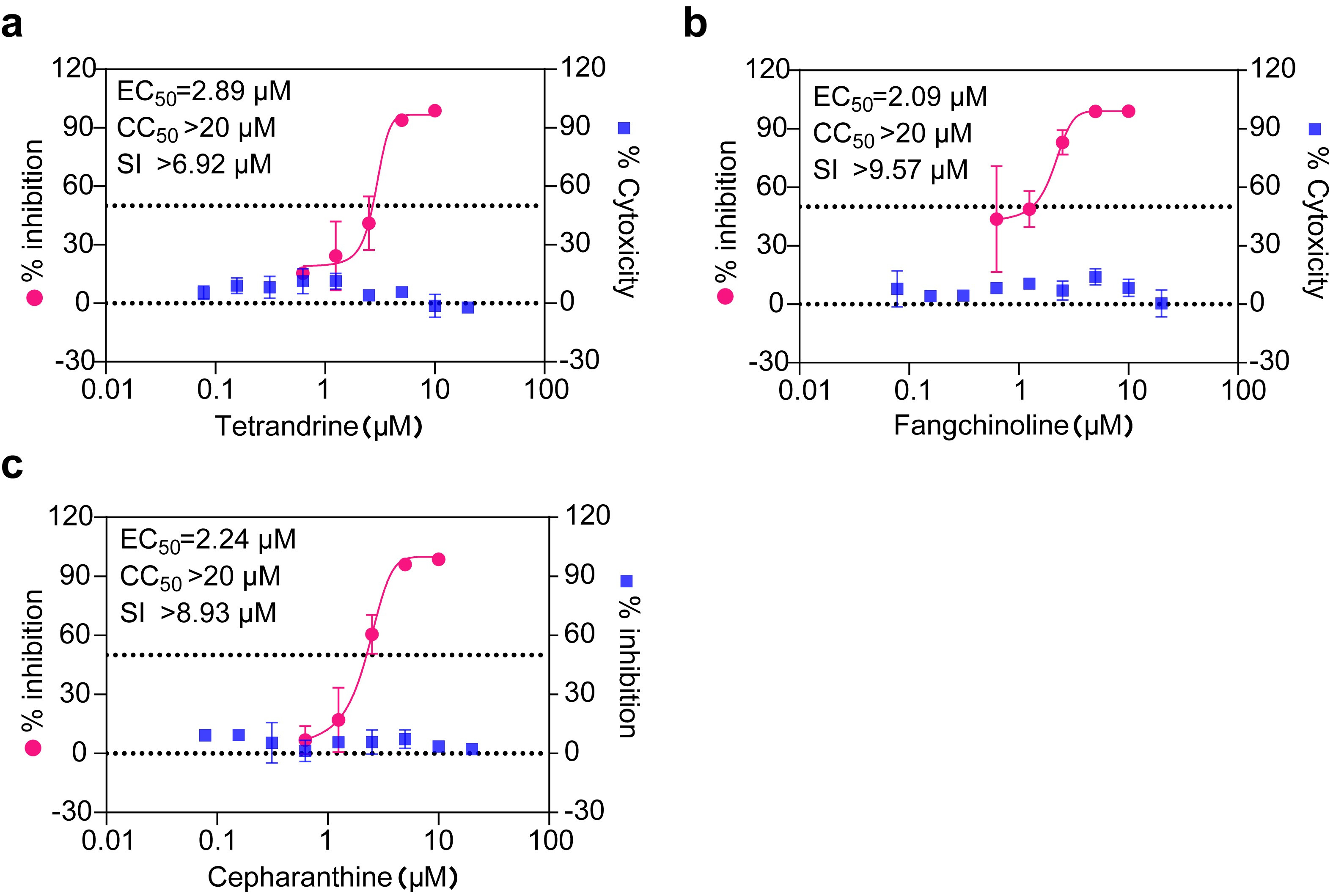

Supplement: S1 Fig — Vero E6 cells were infected with SARS-CoV-2 (MOI = 0.01). After incubation with different concentrations of tetrandrine (a), fangchinoline (b) and cepharanthine (c) at 37°C for 36 h, cell culture fluids were harvested for real-time RT-PCR assay. Cytotoxicity was examined by CCK-8 assay. The CC50, IC50, and SI values for each inhibitor are shown. Experiments were performed at least twice with two technical replicas per experiment and the data are presented as the mean ± standard deviation. Error bars represents standard deviation. (TIF) [file pntd.0010363.s001.tif]
