# Supplementary material for: Berbamine hydrochloride potently inhibits SARS-CoV-2 infection by blocking S protein-mediated membrane fusion
Source: PLoS Negl Trop Dis. 2022 Apr 25;16(4):e0010363. doi: 10.1371/journal.pntd.0010363 (PMC9071123; doi:10.1371/journal.pntd.0010363)
Supplement: S1 Table — (DOCX) [file pntd.0010363.s002.docx]

**Table S1**

| **Compounds** | **EC_50_ (μM)** | **CC_50_ (μM)** | **SI** |
| --- | --- | --- | --- |
| Berbamine hydrochloride | 1.73 | 66.88 | 38.6 |
| Tetrandrine | 2.89 | >20 | >6.92 |
| Fangchinoline | 2.09 | >20 | >9.57 |
| Cepharanthine | 2.24 | >20 | >8.93 |

**Table S1. EC_50_, CC_50_, SI values**
